# Supplementary material for: SyConn2: dense synaptic connectivity inference for volume electron microscopy
Source: Nat Methods. 2022 Oct 24;19(11):1367–70. doi: 10.1038/s41592-022-01624-x (PMC9636020; doi:10.1038/s41592-022-01624-x)
Supplement: Supplementary file 1 — Supplementary Texts 1–3, Supplementary Tables 1–2. [file 41592_2022_1624_MOESM1_ESM.pdf]

nature methods

Brief Communication

https://doi.org/10.1038/s41592-022-01624-x

SyConn2: dense synaptic connectivity inference for volume electron microscopy

In the format provided by the authors and unedited

Supplementary Information

Supplementary Texts

Supp. Text 1 - Comparison of cell type prediction performances

The multi-view CMN[1] was trained using 10-fold cross-validation each with three different random seeds, a batch size of 20, initial learning rate of 1e-3 (scheduler step size 750, decay 0.99), a random set of 20 views per cell and random flip augmentation (independently in x and y for each view with probability 0.5). Cell, synapse, mitochondrion and vesicle cloud meshes were used to render the input views (input channels: 4). In addition, the ratios of predicted symmetric synaptic area over the total synaptic area of the entire cell and dendritic compartments were added as scalar input (n_scalar: 2) to the first fully connected layer of the network. We used seven stacked conv. layers (Conv3DLayer) each containing, applied in the listed order, 3D convolutions (shared weights in z), batch normalization, ReLU activation, max-pooling and dropout (rate: 0.08) and three subsequent fully connected layers (FCLayer): Conv3DLayer(input channels: 4, out channels: 20, kernel size: (1, 5, 5), pooling: (1, 2, 2)), Conv3DLayer(20, 30, (1, 5, 5),(1, 2, 2)), Conv3DLayer(30, 40, (1, 4, 4), (1, 2, 2)), Conv3DLayer(40, 50, (1, 4, 4), (1, 2, 2)), Conv3DLayer(50, 60, (1, 2, 2), (1, 2, 2)), Conv3DLayer(60, 70, (1, 1, 1), (1, 2, 2)), Conv3DLayer(70, 70, (1, 1, 1), (1, 1, 1)), FCLayer(input channels: 4200 + n_scalar, output channels: 100), ReLU activation, FCLayer(100, 50), ReLU activation, FCLayer(50, n_classes: 11). The locations for the view generation were found by voxelization of the cell mesh vertices into voxels of ~3 µm edge length (in total M locations). At every location, 4 views were rendered and the final cell prediction was found by majority vote of the $4M / 20$ individual predictions.

The point-based CMNs (50,000 points, 20 µm context, 50x redundancy) achieved an unweighted class average F1-score of 0.956 ± 0.003 (mean ± s.d.; evaluation scheme as in Fig. 1c) on the 10-fold cross validated ground truth, compared to 0.930 ± 0.006 F1-score with the multi-view approach. Training the point model on inputs without myelin information resulted in an F1-score of 0.932 ± 0.009, similar to the multi-view performance which did not incorporate myelin.

Supp. Text 2 - Comparison of subcellular compartment models

The model comparison for dendrite, axon and soma classification was performed on the same manually annotated and skeletonized neurite reconstructions used in[1–3]. In brief, 27 neurites were used for training (axon: 55 % of vertices, dendrite: 14 %, soma: 31 %), 6 for validation (24 %, 14 %, 62 %) and 28 neurites for evaluation (29 %, 43 %, 28 %). Neurites in the training data were only of a single type, i.e. no sample contained e.g. parts of a soma and dendrite at the same time. Unlabeled neurite skeleton nodes in the test set received the label of the next labeled node found using breadth-first search on the skeleton graph.

The training set was split into mesh contexts with a redundancy of 10, meaning that each neurite was fully covered by different contexts 10 times. Imbalances between the three classes (axon, dendrite, soma) were addressed by rebalancing the number of mesh contexts (dendritic contexts were used 4 times, axon contexts just once, soma contexts 3 times).

The model used mesh contexts generated with a radius of 10 µm sampled with 15,000 points. In addition to the augmentations (see "Point cloud model trainings" in Methods), the points from a mesh context were normalized to a unit sphere and centered at their origin. The model contained kernels of 8 points each and the following layer parameters: (1: 16 kernels, 16 neighbors, no reduction), (2: 16, 16, reduction to 1024 points), (3: 32, 8, reduction to 256 points), (4: 32, 4, reduction to 32 points), (5: 32, 8, deconv to 256 points, residual connection to 4), (6: 16, 8, deconv to 1024 points, residual connection to 3), (7: 16, 16, deconv to original point cloud, residual connection to 2), (8: fully connected, residual to 1).
The total number of trainable parameters was 40,171. Group Normalization was applied after each layer (except the fully connected). The point cloud reduction was done as described in[4]. The model was trained until convergence (250 epochs, training time about 17h, training speed 1.56 samples/s) with a batch size of 16. During training, the model performance was screened on the validation set. The final evaluation on the test set was done by averaging over 10 model checkpoints (from epoch 250 to 340, in increments of 10), where each checkpoint reached near perfect scores on the validation set (F1-score >0.99).

For evaluation, each mesh vertex was labeled with the majority of the multiple predictions it received during inference. A vertex had multiple predictions if it was part of multiple mesh contexts. Each skeleton node was labeled by the result of a majority vote on the mesh vertex predictions within the euclidean Voronoi partitioning of the respective node. The remaining, unclassified nodes were labeled with the prediction of the nearest classified neighbor node.
The achieved scores (skeleton node-level, averaged over 10 model checkpoints) are reported in Supp. Table 2.

Supp. Text 3 - Processing steps for time measurement

For the scalability experiments 24 Google Cloud Computing nodes (n1-highmem-32), each with 32 virtual cores (threads), 2 Tesla P100 and 208 GB memory were used.
All processing steps were grouped as follows:

- Data store: mi and vc SO generation, cell SO and SSO generation.
- Synapse extraction: SO generation (cs, sv-syns and putative synapses), feature extraction and RFC classification.
- Synapse enrichment: Spine head volume estimation and consolidation of synapse properties.
- Morphological analysis: Astrocyte prediction and splitting, compartment prediction (multi-view approach used two models: axon, dendrite, soma, *en-passant* bouton, terminal bouton and dendritic shaft, spine neck, spine head, axon/soma; point-based used three models: axon, dendrite, soma and axon, *en-passant* bouton, terminal bouton and dendritic shaft, spine neck, spine head), cell type classification, morphology embeddings.

Supplementary Tables

	SyConn	SyConn2
input data	Sparse manual cell skeletons, raw data	Compatible with dense automated neuron segmentations, raw data
compartment analysis	Hand-designed features,	Learned features, multi-views[1] / point

	skeleton-based, supervised	clouds
cell type analysis	Hand-designed features, skeleton-based, supervised	Learned features, multi-views[1] / point clouds, unsupervised and supervised
visualization	KNOSSOS (desktop)	Neuroglancer (web-based) / KNOSSOS (desktop)

Supp. Table 1 Overview of SyConn2 updates and features.

metric	precision	s.d.	recall	s.d.	F1-score	s.d.
dendrite	0.98	0.015	0.932	0.014	0.955	0.011
axon	0.951	0.016	0.974	0.002	0.962	0.008
soma	0.955	0.001	0.986	0.017	0.97	0.008
unweighted avg.	0.962	0.008	0.964	0.008	0.963	0.008
weighted avg.	0.963	0.008	0.962	0.008	0.962	0.008

Supp. Table 2 Performance evaluation of the point-based compartment classification on a manually labeled test set of cell skeleton nodes. Overall accuracy (mean ± s.d.): 0.962 ± 0.008.

1. Schubert, P. J., Dorkenwald, S., Januszewski, M., Jain, V. & Kornfeld, J. Learning cellular morphology with neural networks. *Nature Communication* **10**, 2736 (2019).
2. Dorkenwald, S. *et al.* Automated synaptic connectivity inference for volume electron microscopy. *Nat. Methods* **14**, 435–442 (2017).
3. Li, H., Januszewski, M., Jain, V. & Li, P. H. Neuronal Subcompartment Classification and Merge Error Correction. in *Medical Image Computing and Computer Assisted Intervention – MICCAI 2020* 88–98 (Springer International Publishing, 2020).
4. Boulch, A. ConvPoint: Continuous convolutions for point cloud processing. *Comput. Graph.* **88**, 24–34 (2020).
