# Supplementary material for: SyConn2: dense synaptic connectivity inference for volume electron microscopy
Source: Nat Methods. 2022 Oct 24;19(11):1367–70. doi: 10.1038/s41592-022-01624-x (PMC9636020; doi:10.1038/s41592-022-01624-x)
Supplement: Supplementary file 2 — Reporting Summary [file 41592_2022_1624_MOESM2_ESM.pdf]

nature portfolio

Corresponding author(s): Jörgen Kornfeld

Last updated by author(s): Jun 25, 2022

Reporting Summary

Nature Portfolio wishes to improve the reproducibility of the work that we publish. This form provides structure for consistency and transparency in reporting. For further information on Nature Portfolio policies, see our Editorial Policies and the Editorial Policy Checklist.

Statistics

For all statistical analyses, confirm that the following items are present in the figure legend, table legend, main text, or Methods section.

n/a	Confirmed	
☐	☒	The exact sample size (*n*) for each experimental group/condition, given as a discrete number and unit of measurement
☒	☐	A statement on whether measurements were taken from distinct samples or whether the same sample was measured repeatedly
☐	☒	The statistical test(s) used AND whether they are one- or two-sided<br>*Only common tests should be described solely by name; describe more complex techniques in the Methods section.*
☒	☐	A description of all covariates tested
☒	☐	A description of any assumptions or corrections, such as tests of normality and adjustment for multiple comparisons
☐	☒	A full description of the statistical parameters including central tendency (e.g. means) or other basic estimates (e.g. regression coefficient) AND variation (e.g. standard deviation) or associated estimates of uncertainty (e.g. confidence intervals)
☐	☒	For null hypothesis testing, the test statistic (e.g. F, t, r) with confidence intervals, effect sizes, degrees of freedom and P value noted<br>*Give P values as exact values whenever suitable.*
☒	☐	For Bayesian analysis, information on the choice of priors and Markov chain Monte Carlo settings
☒	☐	For hierarchical and complex designs, identification of the appropriate level for tests and full reporting of outcomes
☒	☐	Estimates of effect sizes (e.g. Cohen's *d*, Pearson's *r*), indicating how they were calculated

Our web collection on statistics for biologists contains articles on many of the points above.

Software and code

Policy information about availability of computer code

Data collection	No software has been used for data collection.
Data analysis	SyConn 2.0 (https://github.com/StructuralNeurobiologyLab/SyConn), MorphX 0.1 (https://github.com/StructuralNeurobiologyLab/MorphX), python 3.7, slurm 20.02.6, cuda 10.2, pytorch 1.8, open3d 0.9.0, LightConvPoint 0.2, kimimaro 3.0.0, zmesh 0.5.1, elektronn3 alpha, scikit-image 0.18.3, vigra 1.11.1, scipy 1.6.3, elasticluster 1.3.dev28, Neuroglancer 2.19

For manuscripts utilizing custom algorithms or software that are central to the research but not yet described in published literature, software must be made available to editors and reviewers. We strongly encourage code deposition in a community repository (e.g. GitHub). See the Nature Portfolio guidelines for submitting code & software for further information.

Data

Policy information about availability of data

All manuscripts must include a data availability statement. This statement should provide the following information, where applicable:

- Accession codes, unique identifiers, or web links for publicly available datasets
- A description of any restrictions on data availability
- For clinical datasets or third party data, please ensure that the statement adheres to our policy

Due to large storage requirements, the data sets cannot be made available in a repository but are available at syconn.esc.mpcdf.mpg.de for online viewing and inspection. Please contact the authors to obtain a copy upon reasonable request.

Field-specific reporting

Please select the one below that is the best fit for your research. If you are not sure, read the appropriate sections before making your selection.

☒ Life sciences ☐ Behavioural & social sciences ☐ Ecological, evolutionary & environmental sciences

For a reference copy of the document with all sections, see nature.com/documents/nr-reporting-summary-flat.pdf

Life sciences study design

All studies must disclose on these points even when the disclosure is negative.

Sample size	The sample sizes for the analysis of the minimal mito-synapse distances included all neuron reconstructions from the data set as described in the methods.
Data exclusions	Neuron reconstructions were excluded based on minimum compartment size filters as described in the methods.
Replication	The mito-synapse distance analysis was performed on a single connectomic data set and were selected from 100 randomly selected MSN and 38 GP cells.
Randomization	The order of the synapses for the manual evaluation in the mito-synapse distance analysis was shuffled.
Blinding	The annotation of synapses during synapse evaluation for the analysis of the minimal mito-synapse distances was done without knowledge about associated groups (lower/upper half, cell type).

Reporting for specific materials, systems and methods

We require information from authors about some types of materials, experimental systems and methods used in many studies. Here, indicate whether each material, system or method listed is relevant to your study. If you are not sure if a list item applies to your research, read the appropriate section before selecting a response.

Materials & experimental systems

n/a	Involved in the study	
☒	☐	Antibodies
☒	☐	Eukaryotic cell lines
☒	☐	Palaeontology and archaeology
☒	☐	Animals and other organisms
☒	☐	Human research participants
☒	☐	Clinical data
☒	☐	Dual use research of concern

Methods

n/a	Involved in the study	
☒	☐	ChIP-seq
☒	☐	Flow cytometry
☒	☐	MRI-based neuroimaging
